# Oxidative Stress-Related Gene Polymorphisms Are Associated With Hepatitis B Virus-Induced Liver Disease in the Northern Chinese Han Population

**DOI:** 10.3389/fgene.2019.01290

**Published:** 2020-01-08

**Authors:** Ning Ma, Wenxuan Liu, Xiaolin Zhang, Xia Gao, Fengxue Yu, Weiheng Guo, Yanxin Meng, Ping Gao, Jin Zhou, Meina Yuan, Yingjun Mi, Lei Zhang, Sufen Qi, Lu Li, Luyao Wang, Qiao Su, Lei Yang, Dianwu Liu

**Affiliations:** ^1^ Hebei Key Laboratory of Environment and Human Health, Department of Epidemiology and Statistics, School of Public Health, Hebei Medical University, Shijiazhuang, China; ^2^ The Hebei Key Laboratory of Gastroenterology, Division of Gastroenterology, The Second Hospital of Hebei Medical University, Shijiazhuang, China; ^3^ Antenatal Diagnosis Center, The Fourth Hospital of Shijiazhuang, Shijiazhuang, China; ^4^ Hebei Key Laboratory of Environment and Human Health, Department of Social Medicine and Health Care Management, School of Public Health, Hebei Medical University, Shijiazhuang, China; ^5^ School of Basic Medicine, Hebei Medical University, Shijiazhuang, China

**Keywords:** gene polymorphisms, hepatitis B virus, oxidative stress, *CYBA*, *NCF4*, *Nox4*, *GCLM*

## Abstract

Oxidative stress is closely related to the occurrence and development of various diseases such as cancer, diabetes, and cardiovascular and infectious diseases. We identified six critical genetic variants related to oxidative stress, and evaluated their main effects and their interaction effects on hepatitis B virus (HBV)-induced liver diseases. We enrolled 3,128 Han Chinese subjects into five groups: healthy controls, chronic hepatitis B (CHB), liver cirrhosis (LC), hepatocellular carcinoma (HCC), and natural clearance. We then determined the genotypes in each group for *CYBA*-*rs4673*, *NCF4*-*rs1883112*, *NOX4*-*rs1836882*, *rs3017887*, *SOD2*-rs*4880*, and *GCLM*-*rs41303970*, and evaluated the association between these variants and HBV-induced liver diseases. Gene-gene interactions were evaluated using generalized multifactor dimensionality reduction, logistic regression, and four-by-two tables. Significant associations were observed between healthy controls and the CIB group (CHB+LC+HCC). The *CYBA*-*rs4673*AG genotype was associated with a 1.356 rate of susceptibility of HBV-induced liver disease compared to the wild type GG genotype. The *NCF4*-*rs1883112*G allele occurred more frequently in healthy controls than in the CIB group in all three models (dominant, codominant, and recessive). *Nox4*-*rs1836882* TC showed a protective association, being more frequent in healthy controls compared to the wild type TT genotype. *GCLM*-*rs41303970*A was associated with HBV-induced liver disease. The overall best model by multifactor dimensionality reduction was a five factor interaction model that had the highest cross validation consistency (10/10) and test accuracy (0.5669), *P* *=* 0.001. Oxidative stress-related gene polymorphisms are likely to be associated with HBV-induced liver disease, suggesting that information on these variations is useful for risk assessment of HBV-induced liver disease.

## Introduction

Oxidative stress (OS) is the process by which the body is stimulated by harmful exogenous or endogenous factors (Patterson et al., 2019). Reactive oxygen species (ROS)-induced oxidative DNA damage can also increase chromosomal aberrations that are closely related to the occurrence and development of infectious diseases, cancer, diabetes, and cardiovascular diseases (Fetoni et al., 2019).

There is evidence that hepatitis B virus (HBV) can induce a pro-oxidative state. Sulfhydryl and lipid peroxidation are generated in CHB patients by increasing the OS level (Duygu et al., 2012). In addition, ROS products are increased in carriers with high HBV DNA titers (Fujita et al., 2008). *In vitro* and *in vivo* experiments confirm that HBV infection can induce OS occurrence. A similar increase is also found in chronic hepatitis B (CHB) patients who have significantly higher total peroxide levels, which is considered a parameter of OS, compared to asymptomatic carriers indicate that OS plays a critical role in hepatic injury (Ha et al., 2010). These findings indicate that OS, which is provoked in patients with viral risk factors including HBV, plays critical roles in the pathogenesis of HBV-induced liver diseases such as CHB, liver cirrhosis (LC), and even hepatocellular carcinoma (HCC) (Xianyu et al., 2018). Susceptibility to OS is partly determined by genetic background, also twin studies and segregation analysis support the role of genetic polymorphisms in impacting the host response to HBV infection. Therefore it may be beneficial to detect whether the following gene polymorphisms that regulate key oxidation-reduction enzymes affect the occurrence of HBV-related liver disease.

Nicotinamide adenine dinucleotide phosphate (NADPH) oxidases, also known as nitrogen oxides (Nox), consist of Nox1, Nox2, Nox3, Nox4, Nox5, dual oxidase 1 (Duox1), and Duox2; these oxides transfer electrons through membranes and catalyze the NADPH-dependent reduction of O_2_ to O_2_^−^ to produce ROS (Choi et al., 2014). NADPH oxidase consists of six different subunits, a catalytic core formed by p22phox (encoded by the *CYBA* gene) and Nox2, and four cytoplasmic subunits responsible for activation: p40phox (encoded by the *NCF4* gene), p47phox, p67phox, and rac2 (encoded by *RAC2* gene). Of the catalytic NADPH oxidase subunits, Nox4 (Entrez Gene: 50,507) is the most widely distributed isoform. Together with Nox1 and Nox2, Nox4 is expressed in the liver, and the biological functions of all three Nox subunits need to be combined with another membrane-associated protein subunit, p22phox (Altenhofer et al., 2012). The C242T transformation (*rs4673*) in the *CYBA* gene encoding the p22phox protein can regulate NADPH oxidase activity. The C allele is related to a significant increase in enzyme activity and OS (Tang et al., 2017). Recently, *rs1836882* in the *Nox4* gene promoter region was found to modulate the association between dietary caloric intake and ROS levels in peripheral blood mononuclear cells (PBMCs) (Liu et al., 2013). Another single-nucleotide polymorphism (SNP), *rs3017887* in the *Nox4* gene 5′ UTR region, was chosen as it is the most informative SNP (can capture 12 of 124 SNPs with a minor allelic frequency of at least 0.05) in a haplotype block composed of four SNPs possibly related to oxidative burden (Lim et al., 2009). Another NADPH oxidase polymorphism is 212A>G (*rs1883112*), located in the *NCF4* promoter (encoding p40phox), and is involved in NADPH oxidase down-regulation (Lopes et al., 2004).

ROS clearance in tissues depends on manganese-dependent superoxide dismutase (MnSod), which can catalyze the transformation of active superoxide radicals to hydrogen peroxide and plays an important role in anti-OS response. This mitochondrial enzyme is encoded by the nuclear *SOD2* gene. The substitution of Ala for Val on the 16th amino acid in the signal sequence (Val16Ala, *rs4880*) leads to a decrease in the efficiency of MnSOD entering the mitochondrial matrix and weakens the ability of cells to neutralize superoxide radicals (Wang et al., 2018).

Glutathione (GSH) is the most effective and abundant low molecular weight thiol antioxidant. As a key enzyme in GSH metabolism, the higher expression of glutamate-cysteine ligase (GCL) can significantly reduce the ROS content in tumor cells, and reduce the proliferation rate of tumor cells. GCL is composed of a catalytic subunit (GCLC) and a modifier subunit (GCLM) (Rains and Jain, 2011). The C588T polymorphism (*rs41303970*) in the *GCLM* gene promoter suppresses *GCLM* gene expression, whereas low *GCLM* gene expression is associated with lower plasma GSH levels and enhances OS (Nakamura et al., 2002). In the current study, we tried to clarify whether this polymorphism was involved in HBV predisposition.

Considering the role of OS in the formation and progression of HBV-induced liver diseases, it is important to look for genetic markers capable of identifying individuals at risk for disease progression. Moore (2003) suggests that gene-gene interactions are more important than the independent main effects of any single susceptibility gene, and may synergistically or antagonistically contribute to the increased risk of disease. Therefore, in addition to studying the main effect of these six gene loci, we also verified the interaction effects between them using three different modeling methods.

## Materials and Methods

### Participants

A total of 3,128 unrelated individuals of Han Chinese were recruited from the First, Second, and Fourth Affiliated Hospital of Hebei Medical University and Infectious Disease Hospital of Shijiazhuang City between 2009 and July 2016. The samples included 840 healthy individuals, 691 cases of CHB, 680 cases of LC, 421 HBV-related HCC patients, and 496 HBV natural clearances. The CIB group consisted of the combined values from the CHB, LC, and HCC groups. All participants self-reported as living in the Hebe province of China for 6 months or more.

Healthy individuals were free of hepatitis B surface antigen (HBsAg), anti-HBs, antibodies to hepatitis B core (anti-HBc), and other HBV biomarkers, and normal biochemical parameters as validated by routine blood examinations. They possessed no history of cardiovascular events, diabetes, liver- and kidney-associated diseases, or other autoimmune diseases. HBV natural clearances were defined as being negative for HBsAg plus anti-HBs positive, or positive for both anti-HBs and anti-HBc based on normal liver function. Patients (including CHB, LC, and HCC) were diagnosed by being HBsAg positive, anti-HBc positive, and anti-HBe or HBeAg positive for 6 months or more. The diagnostic criteria for CHB was positivity for HBV-DNA and elevation of alanine aminotransfease (ALT) or aspartate aminotransferase (AST), a histopathological diagnosis of liver injury either by ultrasonography or laboratory testing. LC diagnosis depended on clinical, histological, biochemical, or radiological findings (Levesque et al., 2019). HCC was diagnosed based on magnetic resonance imaging of the liver and/or computed tomography according to published guidelines for HCC diagnosis and management (Sastre et al., 2015). Patients with hepatitis C virus (HCV), alcoholic liver disease, human immunodeficiency virus, or suspected autoimmune diseases were excluded from this study.

The following information was collected for all subjects: basal information (age, gender, history of smoking or drinking, and history of HBV vaccination), disease background (course of the disease, presence or absence of other complications), and biochemistry examination results (five hepatitis B markers, quantity of HBV-DNA, ALT, AST, and AFP concentrations).

The research protocol conforms to the ethical guidelines of the 1975 Declaration of Helsinki and has been approved by the Ethics Committee of Hebei Medical University. All subjects signed an informed consent before being included in the study.

### Selection of Gene Polymorphisms

Candidate SNPs were screened by searching the Ensembl genome browser (http://asia.ensembl.org/index.html), UCSC Genome Browser Home (http://genome.ucsc.edu/), NCBI dbSNP database (http://www.ncbi.nlm.nih.gov/SNP). Criteria for selecting SNPs included: i) SNPs have not been shown to be associated with any HBV-induced disease in Chinese individuals; ii) the minor allele frequency (MAF) of SNPs was greater than 5% in the northern China population; iii) functional SNPs of exons, promoters, or the 3' UTR and 5' UTR regions of genes were preferred; iv) upstream gene variants and splice region variants were also defined as functional SNPs; and v) SNPinfo Web Server was used to predict the function of multiple sites. A total of six SNPs met these criteria (**Table 1**).

**Table 1:** Background information on selected single-nucleotide polymorphisms in Han Chinese people downloaded from 1000 Genome.

Name	Position	Function prediction	MAF	Alleles (minor:major)
rs4673	Exon-missense of CYBA	ESE or ESS/nsSNP	0.073	A:G
rs1883112	Promotor of NCF4	TFBS	0.305	G:A
rs1836882	Promotor of NOX4	TFBS	0.345	C:T
rs3017887	5'UTR of NOX4	TFBS	0.209	A:C
rs4880	Exon-missense of SOD2	ESE or ESS/nsSNP	0.146	G:A
rs41303970	Promotor of GCLM	TFBS	0.180	A:G

The website of 1000 Genome Project: http://www.ensembl.org/index.html?redirect=no.

MAF, minor allele frequency; UTR, untranslated region; TFBS, transcription factor binding sites; nsSNP, nonsynonymous single nucleotide polymorphism; ESE, exonic splicing enhancer; ESS, exonic splicing silencer.

### Genotyping

Two milliliter venous blood samples from all subjects were collected under ethylene diamine tetraacetic acid (EDTA) anticoagulation conditions. Genomic DNA Purification Kits from Promega (San Luis Obispo, CA, USA) were used for genomic DNA extraction, and time of flight mass spectrometry technology (MALDI-TOF MS) from Sequenom Inc. (San Diego, CA, USA) was used for SNP genotyping. Primers for the six SNP alleles were designed by the Bio Miao Biological Company (Beijing, China). Experimental data were processed using MassARRAY Typer 4.0.5 Software (Sequenom). The software determined that more than 97% of the genotyping results of the six SNPs were of conservative (high quality). The genotyping call rate of *rs4673, rs1883112, rs1836882, rs3017887, rs4880*, and *rs41303970* were 99.46, 97.35, 98.95, 99.23, 97.92, and 97.92%, respectively. The genotyping call cluster plots of six SNPs are shown in **Supplementary Figure 1**. For quality control, 1% of each plate was reserved for template positive and negative controls, and repetitive genotyping was randomly selected for 10% of samples. The consistency of two genotyping results reached 100%.

### Statistical Analysis

Statistical analyses were performed using SPSS version 21.0 for windows (SPSS Inc., Chicago, IL, USA). A two-sided *P* value of 0.05 was considered statistically significant in all analyses. Continuous variables of the normal distribution are shown as the mean ± SD. Categorical variables are presented as frequencies. Continuous data were compared using Student's *t*-test and Wilcoxon test. Univariate and multivariate logistic regression analyses were performed to determine the differences in categorical variables between groups. In addition, multivariate analysis was used to correct for confounding factors. To evaluate the influence of SNPs, we set up three models (codominant, dominant, and recessive). Considering the wild type of a SNP as (w) and the variant as (v), then in the codominant model, genotypes w/w, w/v, and v/v were coded as 0, 1, and 2, respectively. Genotype w/w was coded as 0, and w/v and v/v were both coded as 1 in a dominant model. Similarly, when a recessive effect was assumed, w/w and w/v were both scored as 0, and v/v was scored as 1.

Hardy-Weinberg expectations (HWE) were analyzed with SNPStats software (Sole et al., 2006) using the codominant model. Haplotype analysis was conducted by Haploview v4.2 (Barrett et al., 2005). Gene-gene interactions were initially analyzed by the multifactor dimensionality reduction (MDR) algorithm, which was applied using the generalized MDR (GMDR) software package (v0.9). The statistical significance of the MDR models was evaluated by permutation testing. Multiplicative interaction effects were examined with multiple logistic regression analysis. The model included two polymorphisms (two-way) and their interactive term to calculate the *P*-value and odds ratio (*OR*) for each gene-gene interaction. For better interpretation of the results, we defined each genotype as a binary variable (risk genotype *versus* non-risk genotype). Additivity interaction was evaluated using a four-by-two table. Like the variable assignment method of the multiplicative model, risk genotypes and non-risk genotypes were classified into two categories. Three effect indices were used to evaluate the additive model: Synergy index (S), attributable proportion of interaction (AP), and relative excess risk of interaction (RERI). Detailed description of the calculation methods for the three indices are described by Andersson et al. (2005). In the absence of interaction, 95% CI of RERI and AP include 0, 95% CI of S include 1.

## Results

### Demographic Characteristics of the Samples and Candidate Single-Nucleotide Polymorphism Information

**Supplementary Table 1** describes the demographic characteristics and laboratory parameters of the patients and control subjects. There were significant differences in age, sex, smoking habits, and alcohol consumption among the groups. There were also significant differences in the course of the disease, and the HbeAg positive rate between the three patient groups (*P* < 0.001). **Table 1** lists the background information of the candidate SNPs in our study, including the loci position, function prediction, MAF, and mutation base. According to the HWE test (**Table 2**), there was no difference between the observed genotype distribution and the expected distribution of the six SNPs. The MAF values of these six SNPs were consistent with the frequencies listed in the Ensembl database.

**Table 2:** Test of Hardy-Weinberg Equilibrium for the six single-nucleotide polymorphisms in healthy individuals.

	Genotype	Number of subjects	MAF	*χ* ^2^	*P*
rs4673	GG	725	0.072	3.691	0.055
	AG	104			
	AA	8			
rs1883112	AA	351	0.350	0.818	0.366
	AG	360			
	GG	106			
rs1836882	TT	387	0.319	0.109	0.742
	TC	368			
	CC	83			
rs3017887	CC	505	0.218	1.400	0.236
	CA	297			
	AA	34			
rs4880	AA	558	0.171	2.981	0.084
	AG	247			
	GG	17			
rs41303970	GG	572	0.158	0.772	0.380
	AG	223			
	AA	17			

MAF, minor allele frequency.

### Association of Susceptibility of Hepatitis B Virus-Induced Liver Disease and Candidate Single-Nucleotide Polymorphisms

**Table 3** lists the distribution of candidate SNPs genotypes in the CIB and healthy groups. Adjusted logistic regression analysis of factors including age, gender, smoking habits, and alcohol consumption suggested that *rs4673* in *CYBA* was correlated with susceptibility to HBV-induced liver disease. In the codominant genetic model, AG genotype carriers showed a higher risk of HBV-induced liver disease than GG genotype carriers (*OR*: 1.356, *P =* 0.016), with the same relationship (*OR*: 1.315, *P =* 0.026) in the dominant genetic model. Using multivariable logistic analysis, the protective association of the *NCF4*-*rs1883112*G allele was more frequent in healthy controls than in the CIB group in all three models (adjusted *OR =* 0.815, *P =* 0.027 for AG, *OR =* 0.707, *P =* 0.015 for GG in the codominant model; corrected *OR =* 0.790, *P =* 0.007 in the dominant model; adjusted *OR =* 0.733, *P =* 0.029 in the recessive model). The same beneficial action of *rs1836882*TC on HBV-induced liver disease was shown in the codominant genetic model (adjusted *OR =* 0.798, *P =* 0.014), and in the dominant genetic model (adjusted *OR =* 0.805, *P =* 0.013). *SOD2*-rs*4880*G was significantly associated with susceptibility of HBV-induced liver disease under univariable analysis in the dominant genetic model, but after adjusting for confounders, the loci’s protective effect was not significant (*P =* 0.080). With regards to the *GCLM* gene, compared to reference subjects with the *rs41303970*G allele, carriage of the A allele was associated with a higher risk of HBV-induced liver disease susceptibility (adjusted *OR =* 1.916, *P =* 0.022 in the codominant model, and adjusted *OR =* 1.976, *P =* 0.016 in the recessive model). There was no association detected between *Nox4*-*rs3017887* and HBV-induced liver disease in any models.

**Table 3:** Logistic analysis of predictive factors with regard to the risk of hepatitis B virus-induced liver disease between healthy individuals and CIB group.

Loci	Healthy	CIB	Univariable analysis		Multivariable analysis	
			*P*	OR (95% CI)	*Pc*	OR (95% CI)
CYBA-rs4673						
GG	725	1,481		1		1
AG	104	287	0.014*	1.351 (1.061, 1.720)	0.016*	1.356 (1.058, 1.737)
AA	8	12	0.499	0.734 (0.299, 1.804)	0.606	0.785 (0.314, 1.966)
GG/AG+AA	725/112	1,481/299	0.025*	1.307 (1.034, 1.652)	0.026*	1.315 (1.033, 1.674)
GG+AG/AA	829/8	1,768/12	0.440	0.703 (0.286, 1.727)	0.541	0.751 (0.300, 1.879)
						
NCF4-rs1883112						
AA	351	871		1		1
AG	360	719	0.016*	0.805 (0.674, 0.961)	0.027*	0.815 (0.679, 0.978)
GG	106	178	0.005*	0.677 (0.516, 0.887)	0.015*	0.707 (0.534, 0.934)
AA/AG+GG	351/466	871/897	0.003*	0.776 (0.656, 0.917)	0.007*	0.790 (0.666, 0.939)
AA+AG/GG	711/106	1,590/178	0.028*	0.751 (0.581, 0.970)	0.029*	0.733 (0.521, 0.911)
						
NOX4-rs1836882						
TT	387	899		1		1
TC	368	711	0.037*	0.832 (0.699, 0.989)	0.014*	0.798 (0.668, 0.955)
CC	83	165	0.292	0.856 (0.641, 1.143)	0.241	0.836 (0.620, 1.128)
TT/TC+CC	387/451	899/876	0.033*	0.836 (0.709, 0.986)	0.013*	0.805 (0.680, 0.955)
TT+TC/CC	755/83	1,610/165	0.620	0.932 (0.706, 1.230)	0.608	0.928 (0.697, 1.236)
						
NOX4-rs3017887						
CC	505	1,050		1		1
CA	297	629	0.836	1.019 (0.856, 1.212)	0.910	0.990 (0.827, 1.184)
AA	34	95	0.153	1.344 (0.896, 2.016)	0.180	1.330 (0.877, 2.018)
CC/CA+AA	505/331	1,050/724	0.554	1.052 (0.889, 1.244)	0.784	1.025 (0.862, 1.218)
CC+CA/AA	802/34	1,679/95	0.158	1.335 (0.894, 1.992)	0.169	1.335 (0.885, 2.015)
						
SOD2-rs4880						
AA	558	1,266		1		1
AG	247	469	0.057	0.837 (0.697, 1.005)	0.131	0.864 (0.715, 1.044)
GG	17	26	0.209	0.674 (0.363, 1.252)	0.129	0.607 (0.319, 1.156)
AA/AG+GG	558/264	1,266/495	0.037*	0.826 (0.691, 0.989)	0.080	0.847 (0.704, 1.020)
AA+AG/GG	805/17	1,735/26	0.274	0.710 (0.383, 1.315)	0.164	0.634 (0.334, 1.204)
						
GCLM-rs41303970						
GG	572	1,277		1		1
AG	223	422	0.088	0.848 (0.701, 1.025)	0.254	0.892 (0.734, 1.085)
AA	17	62	0.075	1.634 (0.947, 2.819)	0.022*	1.916 (1.097, 3.344)
GG/AG+AA	572/240	1,277/484	0.277	0.903 (0.752, 1.085)	0.691	0.962 (0.796, 1.163)
GG+AG/AA	795/17	1,699/62	0.051	1.707 (0.991, 2.938)	0.016*	1.976 (1.135, 3.440)

CIB, chronic infection with HBV (CHB+LC+HCC).

Pc values were calculated by multivariable analysis controlling for age, sex, alcohol, and tobacco consumption as covariates.

*P < 0.05. Significant results (P value < 0.05) were indicated in bold.

*Rs1836882* and *rs3017887* are located on the same chromosome and, therefore, their close physical location resulted in linkage disequilibrium (LD), which required haplotype block analysis to determine their relationship to susceptibility of HBV-induced liver disease. **Supplementary Figure 2** illustrates that they were in weakened LD. Three haplotypes, ht1(CT), ht2(CC), ht3(AT), accounted for 100% of the distribution and are shown in **Table 4**. The protective association of the CT haplotype was more frequent in the control group than the CIB group, but the difference was not significant (*P =* 0.063).

**Table 4:** Haplotype analysis for two SNPs within *Nox4* gene between CIB and healthy individuals by Haploview.

Haplotype	Freq.	Case, control ratios	*P* value	OR (95% CI)
Block 1				
CT	0.472	1,700.7:1,873.3, 778.3:901.7	0.384	1.053 (0.937, 1.183)
CC	0.301	1,048.2:2,525.8, 534.8:1,145.2	0.063	0.888 (0.783, 1.007)
AT	0.227	825.1:2,748.9, 366.9:1,313.1	0.318	1.074 (0.934, 1.234)

CIB, chronic infection with HBV (CHB+LC+HCC).

Two SNPs, including *rs3017887*_A and rs1836882_C were in almost absolute LD.

### Association of Hepatitis B Virus Natural Clearance and Candidate Single-Nucleotide Polymorphisms

**Supplementary Table 2** lists the distribution of the six SNP genotypes for the CIB and natural clearance groups. With the CIB subjects as cases, and natural clearance individuals as a control group, we found no significant genotypic differences in the six SNP alleles.

### Association of Hepatitis B Virus-Induced Liver Disease Progression and Candidate Single-Nucleotide Polymorphism

**Supplementary Table 3** lists the genotype distribution of the six SNPs for the CHB and LC groups. No positive results were obtained. **Supplementary Table 4** shows the distribution of genotypes for the HCC group and CHB+LC group. These SNPs were ineffective as a tool to differentiate HCC from CHB and LC in the northern Chinese Han population.

### Gene-Gene Interaction in Susceptibility of Hepatitis B Virus-Induced Liver Disease

In the above analysis, almost all candidate SNPs were associated with susceptibility of HBV-induced liver disease, but not with HBV clearance and disease progression. Therefore, interaction analysis was performed only in the CIB and healthy control groups. In the GMDR analysis, all three genotypes of the six SNPs were assigned to 0, 1, 2, according to their pathogenic risk. **Table 5** shows the results of GMDR analysis: the overall best model by MDR was the five-factor interaction model that had the highest cross validation consistency (10/10) and test accuracy (0.5669), *P =* 0.001. The risk of HBV-induced liver disease in the “high-risk” population was 2.221 (1.384, 3.341) times that of the “low-risk” population. **Supplementary Figure 3** presents the distribution of high-risk and low-risk genotypes in the best five-locus model. **Supplementary Table 5** lists the results of the multiplicative interaction analysis of SNPs with HBV-induced liver disease by logistic regression. We found no interaction between the 15 pairs of loci. However, a trend of multiplicative interaction was detected between *rs1883112* and *rs1836882* (*P* = 0.069) that was consistent with the results of the GMDR analysis. Similarly, the additive model did not find any significant interaction terms (**Supplementary Table 6**: 95% CI of RERI and AP included 0, 95% CI of S included 1).

**Table 5:** Generalized multifactor dimensionality reduction models of multi-genes of hepatitis B virus-induced liver disease.

Model	TBA	Sign test (*P*)	CVC
		*(P)*	
rs1883112	0.5139	0.0421*	8/10
rs1883112 rs1836882	0.5257	0.0547	9/10
rs1883112 rs1836882 rs4880	0.5243	0.0547	5/10
rs1883112 rs1836882 rs4880 rs3017887	0.5668	0.0010*	9/10
rs1883112 rs1836882 rs4880 rs41303970 rs3017887**^#^**	0.5669	0.0010*	10/10

TBA, testing balanced accuracy; CVC, cross-validation consistency; **^#^**, the best model.

P values were calculated by GMDR models controlling for age, sex, alcohol, and tobacco consumption as covariates.

*P < 0.05. Significant results (P value < 0.05) were indicated in bold.

## Discussion

Although OS susceptibility is partly determined by genetic background, few studies have been performed on the relationship between functional gene polymorphisms of key enzymes regulated by redox and the risk of HBV-induced liver disease. Consequently, identifying the missing heritability of key enzymes regulated by redox has become a challenging task for HBV-induced liver disease research. In addition, genetic interaction is another significant source of missing heredity. Based on the above hypothesis, we initially assessed the relationship between six genetic polymorphisms related to OS and HBV-induced liver disease in 3,128 samples through four case-control studies.

The exon-missense polymorphism of *CYBA rs4673* results in a substitution of histidine for tyrosine and an alteration of NADPH activity by destroying the heme binding site (Mata-Balaguer et al., 2004). *Rs4673* has been widely demonstrated to be related to coronary artery disease (CAD) (Mazaheri et al., 2017), metabolic disorders (Snahnicanova et al., 2018), and cancer (Megias-Vericat et al., 2018). The association between this locus and HBV-induced liver disease has been studied here for the first time. We therefore inferred the soundness of our results from the function of this locus. Bioinformatics analysis (Mazaheri et al., 2017) indicates a significant impact of *rs4673* on the structure and function of the p22phox protein. It was demonstrated that substitution of tyrosine by histidine on codon 72 of p22phox protein results in a functional mutated protein. Hydrophobicity analysis shows that the hydrophobicity of codon 72 decreases from −0.311 to −0.522 after Y72H substitution. The proportion of sheet structures decreases from 57.9 to 55.4% in the mutated protein. However, the *rs4673* polymorphism has no effect on the structure of the messenger (mRNA). Several teams have researched the biological function of this locus. A study found that carriers of the T(A) allele have reduced enzymatic activity both in stimulated human blood vessel cells and basal cells (Guzik et al., 2000). However, another *in vitro* test showed that the mutant T(A) allele produces higher levels of ROS (Shimo-Nakanishi et al., 2004). Schirmer et al. (2008) found no changes in enzymatic activity owing to mutations at these loci. As the functional study results are highly controversial, the relationship between the mutation site and disease is also controversial. Our findings suggest that the AG (TC) genotype carriers had a higher risk of HBV-induced liver disease than GG (CC) genotype carriers, but there was no significant difference between the AA (TT) and GG (CC) genotypes in the two groups. Overall comparison of the A(T) allele with the G(C) allele produced a non-significant risk estimate for HBV-induced liver disease. These results should be verified with a larger sample size in other ethnic groups.

Another NADPH oxidase subunit analyzed in this study was p40phox, which is involved in the down-regulation of NADPH oxidase (Lopes et al., 2004) and is coded by the *NCF4* gene. *Rs1883112* is located in the promoter of *NCF4* and potentially affects gene expression and ROS generation. Schirmer et al. (2008) found that this SNP does not affect *NCF4* mRNA expression in stimulated granulocytes and basal cells. According to generalized estimating equations (GEE) analysis, *NCF4 rs1883112* is an SNP in the *p40phox* gene regulating NAD(P)H oxidase (Quinn and Gauss, 2004; Olsson et al., 2007). It is an independent host factor and can protect the body from infection, blood toxicity, and cardiotoxicity. Gustafson came to a similar conclusion. He proved that *rs1883112* is the representative of nearby SNP, which has a functional effect on NADPH oxidase activity (Gustafson et al., 2014). As with *rs4673*, the effect of this polymorphism in HBV-induced liver disease was unknown prior to the current study. We found that the *rs1883112*G allele was more frequent in healthy controls than the CIB group and thus had a protective effect. The protective effect of the variant G allele has been demonstrated in other diseases, and is related to higher rate of disease-free survival in diffuse large B-cell lymphoma (DLBCL) (Rossi et al., 2009) and aggressive B-cell non-Hodgkin's lymphoma (NHL) (Gustafson et al., 2014). For acute myeloid leukemia (AML) patients, the *rs1883112* variant G allele is related to higher frequency of complete remission (Megias-Vericat et al., 2018). Because the variant allele has been shown to be protective in different diseases, functional studies on this locus are strongly encouraged.

As the most widely distributed isoform of NADPH oxidase subunits, the *Nox4* gene is reported to be associated with a variety of deleterious effects, including cell senescence and apoptosis, angiogenesis, vascular aging, atherosclerosis (Chen et al., 2012), and other malignancies (Fitzgerald et al., 2012). Jiao et al. (2012) found that Nox4 increases ROS generation, reduces insulin gene expression, and increases β-cell apoptosis both in mice and *in vitro*. The relationship between certain liver diseases and *Nox4* has also been confirmed (de Mochel et al., 2010; Choi et al., 2014; Rabelo et al., 2018). *Rs1836882* and *rs3017887* are located in the *Nox4* promoter and the 5' UTR region, respectively, and may alter the transcriptional activity of the *Nox4* gene, thus being included in our research. Liu et al. (2013) suggested that the *Nox4* promoter region carrying *rs1836882*-TT genotype has a higher binding affinity for HNF3γ protein and a lower transcriptional activity than with the other two genotypes. By silencing the *Nox4* gene or over-expressing HNF3γ, compared with the CT or CC genotypes, the level of ROS is more reduced in the PBMC of high calorie dieters with the TT genotype. In our population study, we found that the TC genotype was significantly more frequent in the healthy group than the CIB group compared to the TT genotype. However, there was no statistical difference in the distribution of CC and TT genotypes between the two groups. Overall comparison of the C allele with the T allele produced a non-significant risk estimate for HBV-induced liver disease. These results are inconsistent with Liu's proposed mechanism. The reason may be that the serum samples in Liu's study were all taken from people on high-calorie diets and experiments were not conducted in a large unbiased sample set. McCaughan et al. (2014) found that this locus is associated with new-onset diabetes after renal transplantation. No other research has been performed on this locus. We did not find that *rs3017887* was related to HBV-induced liver disease. Siqueira et al. (2015) reached a similar conclusion, and no association between the locus and fibrosis in HCV patients was found in his study. Rabelo et al. (2018) confirmed that the *rs3017887* locus was not associated with nonalcoholic steato-hepatitis (NASH). Because the samples of these three studies belong to different liver diseases, repetitive experiments using a large number of samples must be carried out in different races.

Superoxide dismutase (SOD) is one of the most important antioxidants in human beings. SOD2 is the only form that has been certified to be critical for the survival of aerobic life. Genetic mutation of *SOD2* was reported to affect SOD activity, of which the most important was rs*4880*. *R*s*4880* has been researched as a possible predisposing factor in several diseases where OS is deemed to play a key role in pathogenesis, including drug induced liver injury (Lucena et al., 2010), non-alcoholic fatty liver disease, cancers, and alcoholic liver diseases (Huang et al., 2016). Several papers report a relationship between this locus and HCV-related cirrhosis and HCC (Nahon et al., 2007; Jin et al., 2011). To our knowledge, this is the first report to study the effect of *SOD2* polymorphism on HBV-related disease. We found that the AA genotype confers a susceptibility to HBV-induced liver disease compared to the AG and GG genotypes in univariable analysis, but there was no difference between the two groups in genotype distribution when assessed using multivariable analysis. The A to G base substitution at *rs4880* results in the conversion of alanine to valine in the leader amino acid sequence responsible for mitochondrial enzyme localization (Crawford et al., 2012). Xu (Xu et al., 2017) found that the A allele is related to a decrease in *SOD2* mRNA and protein levels. Wang et al. (2012) confirmed the down-regulation of SOD2 and GSH peroxidase expression in HBV transgenic mice. Namiduru et al. (2010) also found a decrease in SOD2 activity in patients with chronic hepatitis B and C. The results of these functional studies are consistent with those of our population study. In our next phase of work, bioinformatics analysis will be performed to evaluate the impact of the polymorphism on structure and function of the corresponding protein using PolyPhen-2 and SNAP software. In addition, the impact of the Val16Ala-*SOD2* polymorphism on protein hydrophobicity will be evaluated through the Kyte-Doolittle scale. RNAsnp web server will be used to evaluate the impact of *rs4880* transitions on mRNA structure.

*Rs41303970*-T is located in the promoter region of the *GCLM* gene, suppresses up-regulation of *GCLM* gene expression, and is related to lower plasma GSH levels (Nakamura et al., 2002). Patients with HBV infection showed significantly lower serum GSH levels than healthy individuals (*P* < 0.01). Nakamura confirmed lower promoter activities in cells for *GCLM*-*rs41303970*T allele carriers. Therefore, the functional consequence predicted from the SNP would be lower GCLM protein level in carriers of the variant allele, which we hypothesized may lead to a decrease in GSH synthesis. There is evidence for tissue-specific regulation of *rs41303970*, where the variant allele shows lower expression in all tissues (data was obtained from the GTEx Portal data base). Samples suitable for gene expression analysis will be necessary for further investigation of this hypothesis. Previous studies show that this mutation is associated with the development of a variety of diseases, such as ischemic heart disease, type 2 diabetes (Katakami et al., 2010), and asthma (Polonikov et al., 2007). Although the disease types differ, their conclusions are consistent with ours: *rs41303970*T is the susceptible base for these diseases, and is consistent with the functional studies of this locus.

The MDR method simplifies high-dimensional genetic data into one-dimension, making gene-gene and gene-environmental interactions detection possible in relatively small sample size with high statistical power. The GMDR method used in our study has the advantage of controlling confounding factors compared to the conventional MDR method. The interaction of testing SNPs using GMDR showed a five factor model with a cross validation consistency of 10 as the strongest risk predictor to HBV-induced liver disease. Six SNPs of the five genes we selected were all OS-related variants, the functional significance of these polymorphisms was confirmed by experimental, case-control studies, or cohort studies. In addition, there is a close connection between these genes; for example, the biological functions of Nox4 need to be combined with another membrane-associated protein subunit, p22phox. Therefore, they may influence each other in terms of pathogenicity. We further tested two-way interaction by multiplicative interaction and additive interaction analysis. Tendency toward multiplicative interaction were detected between *NCF4*-*rs1883112* and *Nox4*-*rs1836882* (*P* = 0.069), which was consistent with the two-factor MDR method model (*P* = 0.0547). Researchers are displaying a growing interest in detecting the genetic interactions that may affect HBV infection susceptibility (Bose et al., 2013; He et al., 2015). However, there are still several factors that limit the analysis of genetic interaction. First, at present there is no optimal analysis method, and different analytical models may lead to conflicting results. Secondly, as statistical interactions are not equivalent to biological interactions, genetic interactions are difficult to explain, which may be the biggest limiting factor hindering this series of studies. However, detection of genetic epistasis may provide new insights into the biological and biochemical pathways that illuminate disease mechanisms, and aid in generating new pathogenesis hypotheses.

This is the first study to examine OS-related gene polymorphisms implicated in susceptibility of HBV-induced liver disease, clearance, and progression in the Han Chinese population, and found that *CYBA*-*rs4673*, *NCF4*-*rs1883112*, *Nox4*-*rs1836882,* and *GCLM*-*rs41303970* are associated with HBV-induced liver disease. It is worth noting that the data obtained from this study should be carefully interpreted and we must note several limitations. To verify these relationships and interactions, larger sample sizes in different ethnic groups are needed for further research. In addition, functional validation of statistically significant SNPs is needed to determine their biological significance. Last but not least, we do not know the significance and biological mechanism underlying the interaction with susceptibility of HBV-induced liver disease. It will be interesting to estimate the functional relationship between SNPs identified in the interaction model and to explore their possible effects on HBV-induced liver disease. However, current biological function trials and biochemical information are still limited. We can only conclude that *Nox4*-rs1836882, rs3017887, *NCF4*-rs1883112, *SOD2*-rs4880, and *GCLM*-*rs41303970* have statistically significant interactive functions in the development of HBV-induced liver disease. Despite the above limitations and concerns, the analysis of gene interactions can provide important avenues of research for discovering biological pathways and mechanisms of disease occurrence, thus forming scientific hypotheses (Cordell, 2009).

## Data Availability Statement

All datasets generated for this study are included in the article/**Supplementary Material**.

## Ethics Statement

The studies involving human participants were reviewed and approved by Ethics Committee of Hebei Medical University. Written informed consent to participate in this study was provided by the participants' legal guardian/next of kin.

## Author Contributions

Concept and design: NM, DL, and LY. Writing of article: NM. Statistical analysis: NM and LZ. Specimen collection and questionnaire investigation: XG, FY, WG, PG, JZ, MY, YiM, SQ, and LL. Inputing data: NM, WL, and XZ. SNP sample management: LW, YaM, and QS. Experiments and procedures: NM, WL, and XZ. Financial support: DL and LY.

## Funding

This work was supported by the Project of Science and Technology Department of Hebei Province (172777123), Key Project of Natural Science Foundation of Hebei Province (H2016206576), National Natural Science Foundation of China (81601876), Science and Technology Research Project of Colleges and Universities in Hebei Province (QN2015006), Innovative experiment program for college students of Hebei Medical University (USIP2019211); National Natural Science Foundation of China (No. 81400322).

## Conflict of Interest

The authors declare that the research was conducted in the absence of any commercial or financial relationships that could be construed as a potential conflict of interest.

## Supplementary Material

The Supplementary Material for this article can be found online at: https://www.frontiersin.org/articles/10.3389/fgene.2019.01290/full#supplementary-material
