# Supplementary material for: Oxidative Stress-Related Gene Polymorphisms Are Associated With Hepatitis B Virus-Induced Liver Disease in the Northern Chinese Han Population
Source: Front Genet. 2020 Jan 8;10:1290. doi: 10.3389/fgene.2019.01290 (PMC6960262; doi:10.3389/fgene.2019.01290)
Supplement: Supplementary file 1 [file DataSheet_1.docx]

Supplementary Table 1 Demographic and etiological characteristics of study subjects

| Characteristics | | Healthy individuals | Natural Clearances | CHB | LC | HCC | *P*-value |
| --- | --- | --- | --- | --- | --- | --- | --- |
| Age(year) (Mean±SD) | | 48.15±14.52 | 50.13±15.11 | 45.93±13.21 | 52.70±10.78 | 57.32±8.96 | ＜0.001***** |
| Gender n(%) | M | 454(54.0) | 290(58.5) | 466(67.4) | 470(69.1) | 326(77.6) | ＜0.001***** |
|  | F | 386(46.0) | 206(41.5) | 225(32.6) | 210(30.9) | 94(22.4) |  |
| Smokers n(%) | Y | 179 (21.3) | 134(27.0) | 254(36.8) | 280(41.2) | 247 (58.7) | ＜0.001***** |
|  | N | 661(78.7) | 362(73.0) | 437 (63.2) | 400(58.8) | 174 (41.3) |  |
| Drinkers n(%) | Y | 281(33.4) | 153(30.8) | 335(48.5) | 306(45.0) | 264(62.7) | ＜0.001***** |
|  | N | 559(66.6) | 343(69.2) | 356(51.5) | 374(55.0) | 157(37.3) |  |
| HbeAg(+/-) | | - | - | 445/246 | 349/331 | 152/265 | ＜0.001***** |
| Length of the HBV infection(year) (Mean±SD) | |  |  | 11.53±2.92 | 18.80±3.55 | 20.06±3.87 | ＜0.001***** |

Abbreviations: CHB, chronic hepatitis B; LC, liver cirrhosis; HCC, hepatocellular carcinoma; SD, standard deviation; M, male; F, female; Y, yes; N, no

**P*＜0.05

Supplementary Table 2 Logistic regression analysis of predictive factors for HBV clearance between CIB and natural clearance

|  |  |  |  | Univariable analysis | |  | Multivariable analysis | |
| --- | --- | --- | --- | --- | --- | --- | --- | --- |
| Loci | Clearances | CIB |  | *P* | OR (95%CI) |  | *Pc* | OR (95%CI) |
| CYBA *-* rs4673 |  |  |  |  |  |  |  |  |
| GG | 426 | 1481 |  |  | 1 |  |  | 1 |
| AG | 62 | 287 |  | 0.057 | 1.332(0.991,1.788) |  | 0.085 | 1.301(0.965,1.755) |
| AA | 6 | 12 |  | 0.272 | 0.575(0.215,1.542) |  | 0.353 | 0.620(0.226,1.701) |
| GG/AG+AA | 426/68 | 1481/299 |  | 0.106 | 1.265(0.952,1.681) |  | 0.141 | 1.242(0.931,1.658) |
| GG+AG/AA | 488/6 | 1768/12 |  | 0.237 | 0.552(0.206,1.478) |  | 0.316 | 0.597(0.218,1.636) |
|  |  |  |  |  |  |  |  |  |
| NCF4 *-* rs1883112 |  |  |  |  |  |  |  |  |
| AA | 234 | 871 |  |  | 1 |  |  | 1 |
| AG | 180 | 719 |  | 0.526 | 1.073(0.863,1.334) |  | 0.619 | 1.058(0.847,1.321) |
| GG | 46 | 178 |  | 0.830 | 1.040(0.729,1.482) |  | 0.739 | 1.064(0.741,1.527) |
| AA/AG+GG | 234/226 | 871/897 |  | 0.540 | 1.066(0.868,1.309) |  | 0.591 | 1.059(0.859,1.306) |
| AA+AG/GG | 414/46 | 1590/178 |  | 0.966 | 1.008(0.716,1.418) |  | 0.837 | 1.037(0.732,1.469) |
|  |  |  |  |  |  |  |  |  |
| NOX4*-* rs1836882 |  |  |  |  |  |  |  |  |
| TT | 254 | 899 |  |  | 1 |  |  | 1 |
| TC | 194 | 711 |  | 0.746 | 1.035(0.838,1.279) |  | 0.900 | 1.014(0.818,1.258) |
| CC | 34 | 165 |  | 0.117 | 1.371(0.924,2.034) |  | 0.188 | 1.309(0.877,1.954) |
| TT/TC+CC | 254/228 | 899/876 |  | 0.425 | 1.086(0.887,1.328) |  | 0.589 | 1.058(0.862,1.300) |
| TT+TC/CC | 448/34 | 1610/165 |  | 0.125 | 1.350(0.920,1.982) |  | 0.185 | 1.301(0.881,1.921) |
|  |  |  |  |  |  |  |  |  |
| NOX4- rs3017887 |  |  |  |  |  |  |  |  |
| CC | 268 | 1050 |  |  | 1 |  |  | 1 |
| CA | 194 | 629 |  | 0.077 | 0.828(0.671,1.020) |  | 0.143 | 0.853(0.689,1.055) |
| AA | 32 | 95 |  | 0.198 | 0.758(0.497,1.156) |  | 0.243 | 0.774(0.504,1.190) |
| CC/CA+AA | 268/226 | 1050/724 |  | **0.049*** | 0.818(0.669,0.999) |  | 0.098 | 0.842(0.686,1.032) |
| CC+CA/AA | 462/32 | 1679/95 |  | 0.338 | 0.817(0.540,1.235) |  | 0.372 | 0.826(0.542,1.257) |
|  |  |  |  |  |  |  |  |  |
| *SOD2*- rs4880 |  |  |  |  |  |  |  |  |
| AA | 350 | 1266 |  |  | 1 |  |  | 1 |
| AG | 124 | 469 |  | 0.704 | 1.046(0.830,1.317) |  | 0.496 | 1.085(0.858,1.373) |
| GG | 6 | 26 |  | 0.693 | 1.198(0.489,2.934) |  | 0.932 | 1.040(0.419,2.585) |
| AA/AG+GG | 350/130 | 1266/495 |  | 0.657 | 1.053(0.839,1.320) |  | 0.499 | 1.083(0.860,1.365) |
| AA+AG/GG | 474/6 | 1735/26 |  | 0.711 | 1.184(0.484,2.893) |  | 0.969 | 1.018(0.411,2.525) |
|  |  |  |  |  |  |  |  |  |
| GCLM - rs41303970 |  |  |  |  |  |  |  |  |
| GG | 348 | 1277 |  |  | 1 |  |  | 1 |
| AG | 128 | 422 |  | 0.363 | 0.898(0.713,1.131) |  | 0.500 | 0.923(0.730,1.166) |
| AA | 14 | 62 |  | 0.534 | 1.207(0.668,2.181) |  | 0.401 | 1.294(0.710,2.359) |
| GG/AG+AA | 348/142 | 1277/484 |  | 0.514 | 0.929(0.744,1.159) |  | 0.713 | 0.959(0.765,1.201) |
| GG+AG/AA | 476/14 | 1699/62 |  | 0.473 | 1.241(0.689,2.236) |  | 0.360 | 1.322(0.727,2.402) |
|  |  |  |  |  |  |  |  |  |

Abbreviations: CIB, chronic infection with HBV (CHB+LC+HCC)

*Pc* values were calculated by multivariable analysis controlling for age, sex, alcohol and tobacco consumption as covariates.

**P*＜0.05

Supplementary Table 3 Logistic regression analysis of predictive factors for disease progression between CHB and LC

|  |  |  |  | Univariable analysis | |  | Multivariable analysis | |
| --- | --- | --- | --- | --- | --- | --- | --- | --- |
| Loci | CHB | LC |  | *P* | OR (95%CI) |  | *Pc* | OR (95%CI) |
| CYBA *-* rs4673 |  |  |  |  |  |  |  |  |
| GG | 564 | 558 |  |  | 1 |  |  | 1 |
| AG | 113 | 115 |  | 0.846 | 1.029(0.774,1.368) |  | 0.924 | 1.015(0.751,1.372) |
| AA | 7 | 3 |  | 0.227 | 0.433(0.111,1.684) |  | 0.618 | 0.704(0.177,2.796) |
| GG/AG+AA | 564/120 | 558/118 |  | 0.966 | 0.994(0.751,1.315) |  | 0.999 | 1.000(0.743,1.345) |
| GG+AG/AA | 677/7 | 673/3 |  | 0.224 | 0.431(0.111,1.674) |  | 0.615 | 0.702(0.177,2.787) |
|  |  |  |  |  |  |  |  |  |
| NCF4 *-* rs1883112 |  |  |  |  |  |  |  |  |
| AA | 334 | 327 |  |  | 1 |  |  | 1 |
| AG | 282 | 271 |  | 0.872 | 0.982(0.783,1.230) |  | 0.529 | 1.080(0.850,1.371) |
| GG | 62 | 75 |  | 0.262 | 1.236(0.854,1.788) |  | 0.130 | 1.352(0.916,1.995) |
| AA/AG+GG | 334/344 | 327/346 |  | 0.804 | 1.027(0.830,1.272) |  | 0.291 | 1.129(0.901,1.414) |
| AA+AG/GG | 616/62 | 598/75 |  | 0.224 | 1.246(0.874,1.777) |  | 0.163 | 1.305(0.898,1.897) |
|  |  |  |  |  |  |  |  |  |
| NOX4*-* rs1836882 |  |  |  |  |  |  |  |  |
| TT | 355 | 330 |  |  | 1 |  |  | 1 |
| TC | 268 | 273 |  | 0.426 | 1.096(0.875,1.373) |  | 0.706 | 1.047(0.825,1.327) |
| CC | 58 | 71 |  | 0.153 | 1.317(0.902,1.922) |  | 0.391 | 1.190(0.800,1.772) |
| TT/TC+CC | 355/326 | 330/344 |  | 0.244 | 1.135(0.917,1.405) |  | 0.539 | 1.073(0.857,1.343) |
| TT+TC/CC | 623/58 | 603/71 |  | 0.207 | 1.265(0.878,1.821) |  | 0.432 | 1.167(0.795,1.713) |
|  |  |  |  |  |  |  |  |  |
| NOX4- rs3017887 |  |  |  |  |  |  |  |  |
| CC | 413 | 383 |  |  | 1 |  |  | 1 |
| CA | 234 | 260 |  | 0.115 | 1.198(0.957,1.500) |  | 0.110 | 1.213(0.957,1.536) |
| AA | 35 | 33 |  | 0.948 | 1.017(0.619,1.669) |  | 0.995 | 1.002(0.593,1.692) |
| CC/CA+AA | 413/269 | 383/293 |  | 0.145 | 1.175(0.946,1.458) |  | 0.144 | 1.185(0.944,1.488) |
| CC+CA/AA | 647/35 | 643/33 |  | 0.833 | 0.949(0.582,1.545) |  | 0.784 | 0.930(0.555,1.559) |
|  |  |  |  |  |  |  |  |  |
| *SOD2*- rs4880 |  |  |  |  |  |  |  |  |
| AA | 488 | 483 |  |  | 1 |  |  | 1 |
| AG | 180 | 179 |  | 0.969 | 1.005(0.789,1.280) |  | 0.664 | 0.945(0.732,1.220) |
| GG | 10 | 10 |  | 0.982 | 1.010(0.417,2.449) |  | 0.983 | 1.011(0.396,2.578) |
| AA/AG+GG | 488/190 | 483/189 |  | 0.967 | 1.005(0.793,1.274) |  | 0.678 | 0.948(0.738,1.218) |
| AA+AG/GG | 668/10 | 662/10 |  | 0.984 | 1.009(0.417,2.440) |  | 0.957 | 1.026(0.403,2.610) |
|  |  |  |  |  |  |  |  |  |
| GCLM - rs41303970 |  |  |  |  |  |  |  |  |
| GG | 508 | 471 |  |  | 1 |  |  | 1 |
| AG | 148 | 176 |  | 0.053 | 1.283(0.997,1.650) |  | 0.056 | 1.296(0.994,1.691) |
| AA | 21 | 23 |  | 0.589 | 1.181(0.645,2.163) |  | 0.703 | 1.131(0.600,2.132) |
| GG/AG+AA | 508/169 | 471/199 |  | 0.051 | 1.270(0.999,1.615) |  | 0.060 | 1.275(0.990,1.643) |
| GG+AG/AA | 656/21 | 647/23 |  | 0.733 | 1.110(0.609,2.026) |  | 0.859 | 1.059(0.564,1.987) |

Abbreviations: CHB, chronic hepatitis B; LC, liver cirrhosis

*Pc* values were calculated by multivariable analysis controlling for age, sex, alcohol and tobacco consumption, length of the HBV infection, HBeAg status as covariates.

Supplementary Table 4 Logistic regression analysis of predictive factors for HCC between HCC and patients with CHB and LC

|  |  |  |  | Univariable analysis | |  | Multivariable analysis | |
| --- | --- | --- | --- | --- | --- | --- | --- | --- |
| Loci | CHB+LC | HCC |  | *P* | OR (95%CI) |  | *Pc* | OR (95%CI) |
| CYBA *-* rs4673 |  |  |  |  |  |  |  |  |
| GG | 1122 | 359 |  |  | 1 |  |  | 1 |
| AG | 228 | 59 |  | 0.180 | 0.809(0.593,1.103) |  | 0.111 | 0.763(0.547,1.064) |
| AA | 10 | 2 |  | 0.545 | 0.625(0.136,2.866) |  | 0.792 | 1.238(0.255,6.017) |
| GG/AG+AA | 1122/238 | 359/61 |  | 0.154 | 0.801(0.590,1.087) |  | 0.127 | 0.775(0.559,1.075) |
| GG+AG/AA | 1350/10 | 418/2 |  | 0.574 | 0.646(0.141,2.960) |  | 0.754 | 1.287(0.265,6.249) |
|  |  |  |  |  |  |  |  |  |
| NCF4 *-*rs1883112 |  |  |  |  |  |  |  |  |
| AA | 661 | 210 |  |  | 1 |  |  | 1 |
| AG | 553 | 166 |  | 0.633 | 0.945(0.749,1.193) |  | 0.831 | 1.028(0.800,1.321) |
| GG | 137 | 41 |  | 0.759 | 0.942(0.643,1.380) |  | 1.000 | 1.000(0.663,1.508) |
| AA/AG+GG | 661/690 | 210/207 |  | 0.609 | 0.944(0.758,1.176) |  | 0.856 | 1.022(0.807,1.295) |
| AA+AG/GG | 1214/137 | 376/41 |  | 0.855 | 0.966(0.669,1.396) |  | 0.951 | 0.988(0.665,1.467) |
|  |  |  |  |  |  |  |  |  |
| NOX4*-* rs1836882 |  |  |  |  |  |  |  |  |
| TT | 685 | 214 |  |  | 1 |  |  | 1 |
| TC | 541 | 170 |  | 0.961 | 1.006(0.799,1.267) |  | 0.523 | 0.922(0.718,1.183) |
| CC | 129 | 36 |  | 0.580 | 0.893(0.599,1.333) |  | 0.293 | 0.794(0.517,1.220) |
| TT/TC+CC | 685/670 | 214/206 |  | 0.886 | 0.984(0.791,1.225) |  | 0.367 | 0.897(0.707,1.136) |
| TT+TC/CC | 1226/129 | 384/36 |  | 0.559 | 0.891(0.605,1.312) |  | 0.359 | 0.824(0.544,1.247) |
|  |  |  |  |  |  |  |  |  |
| NOX4- rs3017887 |  |  |  |  |  |  |  |  |
| CC | 796 | 254 |  |  | 1 |  |  | 1 |
| CA | 494 | 135 |  | 0.200 | 0.856(0.676,1.085) |  | 0.434 | 0.903(0.700,1.166) |
| AA | 68 | 27 |  | 0.360 | 1.244(0.780,1.986) |  | 0.430 | 1.227(0.739,2.038) |
| CC/CA+AA | 796/562 | 254/162 |  | 0.375 | 0.903(0.722,1.131) |  | 0.640 | 0.944(0.741,1.203) |
| CC+CA/AA | 1290/68 | 389/27 |  | 0.241 | 1.317(0.831,2.086) |  | 0.342 | 1.274(0.773,2.098) |
|  |  |  |  |  |  |  |  |  |
| *SOD2*- rs4880 |  |  |  |  |  |  |  |  |
| AA | 971 | 295 |  |  | 1 |  |  | 1 |
| AG | 359 | 110 |  | 0.947 | 1.009(0.785,1.295) |  | 0.671 | 0.943(0.720,1.236) |
| GG | 20 | 6 |  | 0.979 | 0.987(0.393,2.482) |  | 0.782 | 0.871(0.327,2.318) |
| AA/AG+GG | 971/379 | 295/116 |  | 0.953 | 1.007(0.788,1.288) |  | 0.642 | 0.939(0.720,1.224) |
| AA+AG/GG | 1330/20 | 405/6 |  | 0.975 | 0.985(0.393,2.470) |  | 0.806 | 0.885(0.334,2.349) |
|  |  |  |  |  |  |  |  |  |
| GCLM - rs41303970 |  |  |  |  |  |  |  |  |
| GG | 979 | 298 |  |  | 1 |  |  | 1 |
| AG | 324 | 98 |  | 0.962 | 0.994(0.766,1.289) |  | 0.953 | 1.009(0.761,1.336) |
| AA | 44 | 18 |  | 0.304 | 1.344(0.765,2.361) |  | 0.281 | 1.404(0.757,2.604) |
| GG/AG+AA | 979/368 | 298/116 |  | 0.780 | 1.036(0.810,1.324) |  | 0.698 | 1.054(0.808,1.375) |
| GG+AG/AA | 1303/44 | 396/18 |  | 0.298 | 1.346(0.769,2.356) |  | 0.281 | 1.401(0.759,2.587) |

Abbreviations: HCC, hepatocellular carcinoma; LC, liver cirrhosis; CHB, chronic hepatitis B

*Pc* values were calculated by multivariable analysis controlling for age, sex, alcohol and tobacco consumption, length of the HBV infection, HBeAg status as covariate

Supplementary Table 5 Multiplicative interaction analysis of SNPs with HBV infection between healthy individuals and CIB group by logistic regression

|  | *B* | *S.E* | *Wald* | *P* | *OR (95%CI)* |
| --- | --- | --- | --- | --- | --- |
| rs4673 | 0.267 | 0.168 | 2.522 | 0.112 | 1.306(0.939,1.815) |
| rs1883112 | 0.227 | 0.095 | 5.743 | 0.017 | 1.255(1.042,1.511) |
| rs4673 by rs1883112 | 0.066 | 0.255 | 0.068 | 0.795 | 1.068(0.649,1.760) |
| Constant | -0.497 | 0.205 | 5.880 | 0.015 | 0.608 |
| rs4673 | 0.192 | 0.164 | 1.376 | 0.241 | 1.212(0.879,1.670) |
| rs1836882 | 0.206 | 0.094 | 4.789 | 0.029 | 1.228(1.022,1.476) |
| rs4673 by rs1836882 | 0.174 | 0.251 | 0.481 | 0.488 | 1.190(0.728,1.946) |
| Constant | -0.520 | 0.205 | 6.412 | 0.011 | 0.595 |
| rs4673 | 0.168 | 0.234 | 0.516 | 0.473 | 1.183(0.748,1.871) |
| rs4880 | 0.132 | 0.102 | 1.667 | 0.197 | 1.141(0.934,1.395) |
| rs4673 by rs4880 | 0.169 | 0.277 | 0.374 | 0.541 | 1.184(0.688,2.038) |
| Constant | -0.449 | 0.209 | 4.619 | 0.032 | 0.638 |
| rs4673 | 0.239 | 0.125 | 3.654 | 0.056 | 1.270(0.994,1.624) |
| rs41303970 | 0.632 | 0.296 | 4.569 | 0.033 | 1.881(1.054,3.359) |
| rs4673 by rs41303970 | 0.488 | 1.118 | 0.190 | 0.663 | 1.629(0.182,14.577) |
| Constant | -0.397 | 0.201 | 3.889 | 0.049 | 0.673 |
| rs1883112 | 0.388 | 0.122 | 10.068 | 0.002 | 1.475(1.160,1.874) |
| rs1836882 | 0.349 | 0.119 | 8.673 | 0.003 | 1.418(1.124,1.790) |
| rs1883112 by rs1836882 | -0.320 | 0.176 | 3.304 | 0.069 | 0.726(0.514,1.025) |
| Constant | -0.630 | 0.213 | 8.754 | 0.003 | 0.532 |
| rs1883112 | 0.408 | 0.163 | 6.270 | 0.012 | 1.504(1.093,2.070) |
| rs4880 | 0.270 | 0.126 | 4.616 | 0.032 | 1.310(1.024,1.677) |
| rs1883112 by rs4880 | -0.224 | 0.195 | 1.321 | 0.250 | 0.799(0.546,1.171) |
| Constant | -0.587 | 0.222 | 7.017 | 0.008 | 0.556 |
| rs1883112 | 0.240 | 0.090 | 7.081 | 0.008 | 1.271(1.065,1.517) |
| rs41303970 | 0.569 | 0.400 | 2.018 | 0.155 | 1.766(0.806,3.870) |
| rs1883112by rs41303970 | 0.039 | 0.571 | 0.005 | 0.946 | 1.040(0.340,3.182) |
| Constant | -0.437 | 0.206 | 4.502 | 0.034 | 0.646 |
| rs1836882 | 0.009 | 0.158 | 0.003 | 0.955 | 1.009(0.740,1.376) |
| rs4880 | 0.024 | 0.132 | 0.033 | 0.857 | 1.024(0.790,1.328) |
| rs1836882 by rs4880 | 0.284 | 0.190 | 2.242 | 0.134 | 1.329(0.916,1.928) |
| Constant | -0.429 | 0.222 | 3.732 | 0.053 | 0.651 |
| rs1836882 | 0.221 | 0.089 | 6.121 | 0.013 | 1.247(1.047,1.485) |
| rs41303970 | 0.586 | 0.334 | 3.067 | 0.080 | 1.796(0.933,3.460) |
| rs1836882by rs41303970 | 0.331 | 0.650 | 0.259 | 0.611 | 1.392(0.389,4.983) |
| Constant | -0.470 | 0.207 | 5.147 | 0.023 | 0.625 |
| rs41303970 | 0.138 | 0.480 | 0.082 | 0.774 | 1.148(0.448,2.940) |
| rs4880 | 0.157 | 0.097 | 2.611 | 0.106 | 1.171(0.967,1.417) |
| rs41303970 by rs4880 | 0.955 | 0.620 | 2.370 | 0.124 | 2.598(0.770,8.758) |
| Constant | -0.401 | 0.210 | 3.642 | 0.056 | 0.669 |
| rs3017887 | 0.326 | 0.232 | 1.981 | 0.159 | 1.386(0.880,2.184) |
| rs4673 | 0.291 | 0.127 | 5.250 | 0.022 | 1.338(1.043,1.716) |
| rs3017887 by rs4673 | -0.064 | 0.579 | 0.012 | 0.912 | 0.938(0.302,2.918) |
| Constant | -0.409 | 0.198 | 4.242 | 0.039 | 0.665 |
| rs3017887 | 0.466 | 0.312 | 2.236 | 0.135 | 1.594(0.865,2.939) |
| rs1883112 | 0.268 | 0.090 | 8.803 | 0.003 | 1.307(1.095,1.560) |
| rs3017887 by rs1883112 | -0.399 | 0.422 | 0.892 | 0.345 | 0.671(0.293,1.535) |
| Constant | -0.455 | 0.204 | 5.000 | 0.025 | 0.634 |
| rs3017887 | 19.782 | 40192.7 | .000 | 1.000 | -- |
| rs1836882 | 0.193 | 0.089 | 4.679 | 0.031 | 1.213(1.018,1.445) |
| rs3017887 by rs1836882 | -19.597 | 40192.7 | .000 | 1.000 | -- |
| Constant | -0.453 | 0.203 | 4.959 | 0.026 | 0.636 |
| rs3017887 | 0.778 | 0.411 | 3.589 | 0.058 | 2.177(0.973,4.868) |
| rs4880 | 0.182 | 0.097 | 3.515 | 0.061 | 1.200(0.992,1.452) |
| rs3017887 by rs4880 | -0.638 | 0.483 | 1.748 | 0.186 | 0.528(0.205,1.361) |
| Constant | -0.424 | 0.208 | 4.178 | 0.041 | 0.654 |
| rs3017887 | 0.256 | 0.211 | 1.464 | 0.226 | 1.291(0.853,1.954) |
| rs41303970 | 0.603 | 0.286 | 4.444 | 0.035 | 1.828(1.043,3.203) |
| rs3017887by rs41303970 | 19.952 | 28417.6 | .000 | 0.999 | -- |
| Constant | -0.343 | 0.200 | 2.937 | 0.087 | 0.710 |

Genotype assignment based on the optimum risk association results of genetic models:

rs4673：AG+AA (1), GG(0) ; rs1883112: AA(1), GG +AG(0); rs1836882: TT(1), TC + CC (0);

rs4880: AA(1), GG +AG(0); rs41303970:AA(1), GG +AG(0); rs3017887: AA(1),CC+CA(0)

*P* values were calculated by multivariable analysis controlling for age, sex, alcohol and tobacco consumption as covariates.

Supplementary Table 6 Additive interaction analysis of SNPs with HBV infection between healthy individuals and CIB group

| SNP1 | SNP2 | CIB | Control | Β | *P* | *OR(95%CI)* | *RERI/AP/S* |
| --- | --- | --- | --- | --- | --- | --- | --- |
| rs4673 | rs1883112 |  |  |  |  |  |  |
| GG | GG +AG | 744 | 402 |  |  | 1.0 |  |
| GG | AA | 723 | 307 | 0.227 | 0.017 | 1.255(1.042,1.511) | *RERI:0.190(-0.568,0.948)* |
| AG+AA | GG +AG | 147 | 62 | 0.267 | 0.112 | 1.306(0.939,1.815) | *AP:0.108(-0.296,0.513)* |
| AG+AA | AA | 144 | 43 | 0.560 | 0.003 | 1.751(1.208,2.537) | *S:1.338(0.420,4.262)* |
| rs4673 | rs1836882 |  |  |  |  |  |  |
| GG | CC +TC | 724 | 382 |  |  | 1.0 |  |
| GG | TT | 751 | 341 | 0.206 | 0.029 | 1.228(1.022,1.476) | *RERI:0.331(-0.411,1.074)* |
| AG+AA | CC +TC | 150 | 69 | 0.192 | 0.241 | 1.212(0.879,1.670) | *AP:0.187(-0.183,0.557)* |
| AG+AA | TT | 144 | 43 | 0.572 | 0.003 | 1.771(1.221,2.568) | *S:1.752(0.489,6.277)* |
| rs4673 | rs4880 |  |  |  |  |  |  |
| GG | GG +AG | 416 | 229 |  |  | 1.0 |  |
| GG | AA | 1041 | 482 | 0.132 | 0.197 | 1.141(0.934,1.395) | *RERI:0.274(-0.414,0.963)* |
| AG+AA | GG +AG | 74 | 32 | 0.168 | 0.473 | 1.183(0.748,1.871) | *AP:0.172(-0.238,0.581)* |
| AG+AA | AA | 220 | 76 | 0.469 | 0.003 | 1.599(1.167,2.191) | *S:1.847(0.272,12.528)* |
| rs4673 | rs41303970 |  |  |  |  |  |  |
| GG | GG +AG | 1409 | 682 |  |  | 1.0 |  |
| GG | AA | 52 | 16 | 0.632 | 0.033 | 1.881(1.054,3.359) | *RERI:1.741(-6.516,9.998)* |
| AG+AA | GG +AG | 283 | 110 | 0.239 | 0.056 | 1.270(0.994,1.624) | *AP:0.447(-0.749,1.644)* |
| AG+AA | AA | 9 | 1 | 1.359 | 0.205 | 3.893(0.475,31.898) | *S:2.512(0.126,50.063)* |
| rs1883112 | rs1836882 |  |  |  |  |  |  |
| GG +AG | CC +TC | 424 | 258 |  |  | 1.0 |  |
| GG +AG | TT | 609 | 206 | 0.349 | 0.003 | 1.418(1.124,1.790) | *RERI:-0.373(-0.865,0.120)* |
| AA | CC +TC | 442 | 178 | 0.388 | 0.002 | 1.475(1.160,1.874) | *AP:-0.245(-0.580,0.089)* |
| AA | TT | 423 | 173 | 0.418 | 0.001 | 1.519(1.192,1.935) | *S:0.582(0.305,1.111)* |
| rs1883112 | rs4880 |  |  |  |  |  |  |
| GG +AG | GG +AG | 257 | 165 |  |  | 1.0 |  |
| GG +AG | AA | 629 | 297 | 0.270 | 0.032 | 1.310(1.024,1.677) | *RERI:-0.238(-0.780,0.305)* |
| AA | GG +AG | 227 | 94 | 0.408 | 0.012 | 1.504(1.093,2.070) | *AP:-0.151(-0.492,0.191)* |
| AA | AA | 626 | 243 | 0.455 | 0.0004 | 1.576(1.224,2.028) | *S:0.708(0.361,1.389)* |
| rs1883112 | rs41303970 |  |  |  |  |  |  |
| GG +AG | GG +AG | 852 | 441 |  |  | 1.0 |  |
| GG +AG | AA | 26 | 9 | 0.569 | 0.155 | 1.766(0.806,3.870) | *RERI:0.295(-2.000,2.590)* |
| AA | GG +AG | 829 | 331 | 0.240 | 0.008 | 1.271(1.065,1.517) | *AP:0.126(-0.781,1.034)* |
| AA | AA | 32 | 8 | 0.847 | 0.037 | 2.334(1.052,5.175) | *S:1.284(0.189,8.735)* |
| rs1836882 | rs4880 |  |  |  |  |  |  |
| CC +TC | GG +AG | 248 | 134 |  |  | 1.0 |  |
| CC +TC | AA | 613 | 304 | 0.024 | 0.857 | 1.024(0.790,1.328) | *RERI:0.340(-0.033,0.712)* |
| TT | GG +AG | 241 | 128 | 0.009 | 0.955 | 1.009(0.740,1.376) | *AP:0.247(-0.031,0.525)* |
| TT | AA | 645 | 254 | 0.317 | 0.018 | 1.373(1.055,1.787) | *S:11.191 (0.00,3.02×10^7^)* |
| rs1836882 | rs41303970 |  |  |  |  |  |  |
| CC +TC | GG +AG | 820 | 419 |  |  | 1.0 |  |
| CC +TC | AA | 38 | 13 | 0.586 | 0.080 | 1.796(0.933,3.460) | *RERI:1.073(-2.508,4.654)* |
| TT | GG +AG | 868 | 374 | 0.221 | 0.013 | 1.247(1.047,1.485) | *AP:0.344(-0.461,1.150)* |
| TT | AA | 21 | 4 | 1.137 | 0.042 | 3.119(1.045,9.312) | *S:2.028 (0.286,14.388)* |
| rs4880 | rs41303970 |  |  |  |  |  |  |
| GG +AG | GG +AG | 467 | 247 |  |  | 1.0 |  |
| GG +AG | AA | 1209 | 532 | 0.157 | 0.106 | 1.171(0.967,1.417) | *RERI:2.172(-0.699,5.043)* |
| AA | GG +AG | 15 | 7 | 0.138 | 0.774 | 1.148(0.448,2.940) | *AP:0.622(0.200,1.045)* |
| AA | AA | 44 | 8 | 1.250 | 0.002 | 3.490(1.600,7.611) | *S:7.832 (0.203,302.144)* |
| rs3017887 | rs4673 |  |  |  |  |  |  |
| CC+CA | GG | 1395 | 695 |  |  | 1.0 |  |
| CC+CA | AG+AA | 278 | 105 | 0.291 | 0.022 | 1.338(1.043,1.716) | *RERI: 0.017(-1.879,1.913)* |
| AA | GG | 77 | 28 | 0.326 | 0.159 | 1.386(0.880,2.184) | *AP: 0.010(-1.070,1.090)* |
| AA | AG+AA | 18 | 5 | 0.554 | 0.286 | 1.740(0.629,4.814) | *S:1.024(0.078,13.365)* |
| rs3017887 | rs1883112 |  |  |  |  |  |  |
| CC+CA | GG +AG | 844 | 451 |  |  | 1.0 |  |
| CC+CA | AA | 816 | 328 | 0.268 | 0.003 | 1.307(1.095,1.560) | *RERI:-0.503(-1.744,0.737)* |
| AA | GG +AG | 45 | 15 | 0.466 | 0.135 | 1.594(0.865,2.939) | *AP:-0.360(-1.372,0.653)* |
| AA | AA | 50 | 19 | 0.335 | 0.236 | 1.398(0.803,2.435) | *S:0.442(0.050,3.909)* |
| rs3017887 | rs1836882 |  |  |  |  |  |  |
| CC+CA | CC +TC | 872 | 449 |  |  | 1.0 |  |
| CC+CA | TT | 794 | 351 | 0.193 | 0.031 | 1.213(1.018,1.445) | *RERI:--* |
| AA | CC+TC | 1 | 0 | 19.782 | 1.000 | -- | *AP:--* |
| AA | TT | 94 | 34 | 0.379 | 0.077 | 1.460(0.960,2.222) | *S:--* |
| rs3017887 | rs4880 |  |  |  |  |  |  |
| CC+CA | GG +AG | 454 | 252 |  |  | 1.0 |  |
| CC+CA | AA | 1199 | 534 | 0.182 | 0.061 | 1.200(0.922,1.452) | *RERI:-0.997(-2.871,0.878)* |
| AA | GG +AG | 34 | 8 | 0.778 | 0.058 | 2.177(0.973,4.868) | *AP: -0.722(-2.227,0.782)* |
| AA | AA | 60 | 24 | 0.322 | 0.217 | 1.380(0.828,2.302) | *S:0.276(0.032,2.407)* |
| rs3017887 | rs41303970 |  |  |  |  |  |  |
| CC+CA | GG +AG | 1597 | 759 |  |  | 1.0 |  |
| CC+CA | AA | 57 | 17 | 0.603 | 0.035 | 1.828(1.043,3.203) | *RERI:--* |
| AA | GG +AG | 92 | 34 | 0.256 | 0.226 | 1.291(0.853,1.954) | *AP:--* |
| AA | AA | 2 | 0 | 20.811 | 0.999 | -- | *S:--* |

Abbreviations: CIB, chronic infection with HBV (CHB+LC+HCC)；*RERI*, Relative Excess Risk of Interaction; *AP,* Attributable Proportion of interaction; *S:* Synergy index

When calculating covariance matrix, the covariates such as gender, age, drinking and smoking were controlled

Supplementary Fig.1 The genotyping call cluster plots of six SNPs


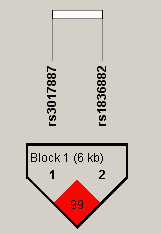


Supplementary Fig.2 Haplotype analysis for 2 SNPs within *NOX4* gene between CIB and healthy individuals by Haploview


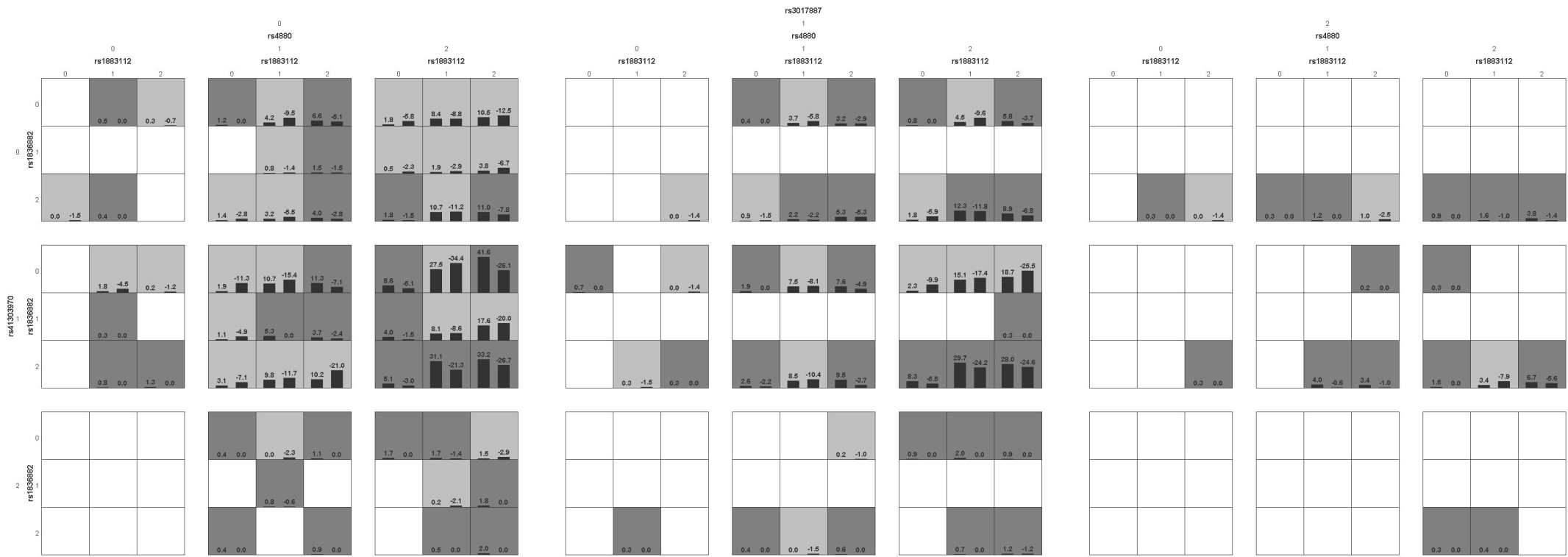


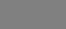
High risk
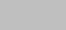
 Low-risk
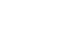
 Empty

Supplementary Fig 3. Distribution of high-risk (Dark shading) and low-risk (Light shading) genotypes in the best five-locus model. The scores of CIB group (left black bar in boxes) and control group (right black bar in boxes) are shown for each genotype combination.
